# Epidermal growth factor receptor-regulated miR-125a-5p – a metastatic inhibitor of lung cancer

**DOI:** 10.1111/j.1742-4658.2009.07238.x

**Published:** 2009-10

**Authors:** Guofu Wang, Weimin Mao, Shu Zheng, Jingjia Ye

**Affiliations:** 1 Department of Respiratory Disease, Zhejiang HospitalHangzhou, China; 2 College of Life Sciences, Zhejiang UniversityHangzhou, China

**Keywords:** epidermal growth factor receptor, lung neoplasm, metastasis, microRNA, miR-125a

## Abstract

Both the epidermal growth factor receptor signaling pathway and microRNA (miRNA) play an important role in lung cancer development and progression. To address the potential role of miRNA in epidermal growth factor receptor signaling, we identified miR-125a-5p as a downstream target, using an miRNA array. We further demonstrated that miR-125a-5p inhibited migration and invasion of lung cancer cells. Moreover, miR-125a-5p regulated the expression of several downstream genes of epidermal growth factor receptor signaling. Importantly, examination of lung cancer samples revealed a significant correlation of miR-125a-5p repression with lung carcinogenesis. Taken together, our results provide compelling evidence that miR-125a-5p, an epidermal growth factor-signaling-regulated miRNA, may function as a metastatic suppressor.

## Introduction

Lung cancer is the most frequent cause of cancer death in the USA, with a mortality of approximately 85% for all stages, according to population statistics [1]. Furthermore, it is the most common cancer both in incidence rate and in death rate in developing countries such as China [2]. Clinical data have shown that most lung cancer patients eventually suffer relapse and/or metastasis after complete excision of the cancer, even if they were at stage IA [3]. Despite the progress that has been made in recent decades, the mechanism of lung cancer development, including relapse and metastasis, is not fully understood.

Growth factor signal transduction pathways play key roles in various physiological and pathological processes, encompassing metabolism, growth, proliferation, stress, development, and apoptosis. Abnormalities in these signaling pathways lead to various developmental disorders and diseases. In severe cases, aberrant growth factor signaling may even give rise to tumors. Among these pathways, epidermal growth factor receptor (EGFR) signaling appears to be particularly important for epithelial malignancies, including lung cancer [4]. However, despite the clinical importance, the underlying molecular mechanism by which EGFR signaling regulates lung cancer development remains poorly understood.

Recent studies have indicated that microRNAs (miRNAs) are extensively involved in various signaling pathways [5–7]. MicroRNAs are a class of small, noncoding RNAs that play important roles in different biological processes. Interestingly, since Calin *et al.* first reported that miR-15 and miR-16 are deleted or downregulated in the majority (approximately 68%) of chronic lymphocytic leukemia cases [8], accumulating evidence has implicated miRNA in human cancer [9]. Additionally, altered expression of miRNA has been shown to mediate tumor metastasis [10,11]. However, the relationship between miRNA and EGFR signaling remains largely elusive.

Here, we set out to characterize the regulation of miRNA expression by EGFR activation, using microarray analysis. We further determined that miR-125a-5p could negatively regulate lung cancer cell migration and invasion *in vitro*, and that this was frequently decreased in lung cancer patients. Our data strongly implicate miR-125a-5p as a potential inhibitor of tumor metastasis.

## Results

### Repression of miR-125a-5p in response to EGFR activation in lung cancer cells

**Fig. 1:**
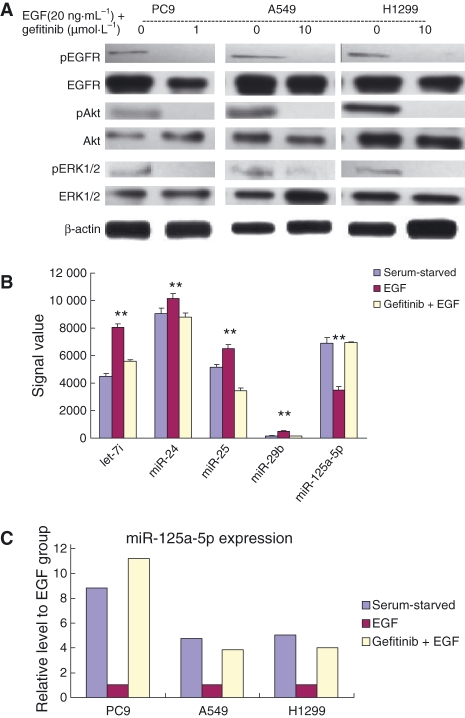
Gefitinib inhibited EGF-induced EGFR, ERK1/2 and Akt phosphorylation and reversed EGF-stimulated miRNA expression in lung cancer cells. (A) Western blot showed that, after EGF (20 ng·mL^−1^) stimulation, phosphorylation of EGFR, ERK1/2 and Akt occurred in PC9, A549 and H1299 lung cancer cells, and that this could be almost completely abolished by gefitinib at different concentrations. (B) After EGF stimulation, miRNA microarray analysis revealed that the expression of 39 miRNAs was significantly altered (*P* < 0.01). Among these, five miRNAs, let-7i, miR-24, miR-25, miR-29b, and miR-125a-5p, were further confirmed as EGFR-regulated miRNAs by gefitinib treatment. **P < 0.01 as compared with the serum-starved medium group. (C) Quantitative RT-PCR showed that the mir-125a-5p level was significantly reduced after EGF (20 ng·mL^−1^) stimulation in all three cell lines, and that this effect was reversed by gefitinib. The value for miR-125a-5p in the EGF group was set at 1, and the relative amounts of miR-125a-5p in the other groups were plotted as fold induction.

It has been well established that EGFR signaling plays a crucial role in lung tumorigenesis. Interestingly, accumulating evidence has now implicated miRNA in the formation and malignant progression of human cancer. To examine the potential relationship between EGFR signaling and miRNA, we first performed miRNA expression profiling after epidermal growth factor (EGF) stimulation using the miRHuman_10.0_070802 miRNA array, which contains 711 probes. Through comparison of RNA prepared from a combination of three lung cells, A549, PC9, and H1299, before or after EGF stimulation, our profiling analysis revealed 39 miRNAs with significantly different expression levels (*P* < 0.01; Table 1). To further determine the requirement for EGFR signaling in the expression of miRNA listed in Table 1, we blocked EGFR signaling with gefitinib. Gefitinib is an inhibitor of tyrosine kinase that competes with ATP for binding to the intracellular kinase domain, preventing receptor activation and the engagement of downstream signaling transducers [12]. Thus, it has been widely used to interfere with EGFR signaling. Consistent with previous observations [13,14], PC9 cells, which express a mutant EGFR and have been extensively explored before, were more sensitive to gefitinib. Thus, a low concentration of gefitinib could abolish the phosphorylation of EGFR, extracellular signal-related kinase (ERK)1/2 and Akt after EGF stimulation. However, H1299 and A549 cells expressed wild-type EGFR, and were less sensitive to gefitinib. Only a high concentration of gefitinib could decrease the phosphorylation of EGFR, ERK1/2, and Akt after EGF stimulation (Fig. 1A). Accordingly, our miRNA array analysis showed that, among the 39 miRNAs listed in Table 1, five miRNAs, inducing let-7i, miR-24, miR-25, miR-29b, and miR-125a-5p, could be reversed by gefitinib (Fig. 1B).

**Table 1:** MicroRNA array analysis showed 39 miRNAs were in response to EGF stimulation in lung cancer cells (*P* < 0.01).

Name	Prestimulation	Poststimulation	Log ratio
miR-542-5p	51	228	3.19
miR-29b	169	506	1.57
miR-663	621	1125	1.16
let-7i	4494	8048	0.47
miR-25	5162	6490	0.43
miR-19b	909	1077	0.37
miR-29a	10 713	12 060	0.29
miR-15a	538	596	0.26
miR-24	9069	10 155	0.21
miR-17	6205	5218	−0.25
miR-106a	5990	4989	−0.28
miR-455-3p	303	263	−0.32
miR-151-5p	6483	5093	−0.34
miR-484	299	238	−0.35
miR-23a	21 099	17 298	−0.36
miR-181b	1309	1207	−0.41
miR-30d	1661	1203	−0.43
miR-26a	11 565	9160	−0.44
miR-361-5p	3379	2631	−0.44
miR-183	1536	1267	−0.51
miR-23b	21 710	15 380	−0.54
miR-15b	11 620	8384	−0.64
miR-130a	315	255	−0.64
miR-331-3p	272	164	−0.65
miR-99b	2920	1896	−0.69
let-7a	21 505	13 477	−0.80
miR-30c	5450	3314	−0.81
miR-224	2871	2090	−0.87
miR-30b	4382	2658	−0.92
let-7f	16 236	10 547	−0.94
miR-125a-5p	6882	3518	−1.03
let-7e	9822	5648	−1.06
let-7d	14 071	8423	−1.08
let-7c	13 905	7087	−1.21
miR-200c	7237	6774	−1.23
miR-574-5p	666	410	−1.28
miR-342-3p	394	174	−1.29
let-7b	7853	3379	−1.48
miR-122	5148	254	−3.54

We further verified our array results by quantitative PCR, which revealed the expression of miR-24, miR-25, miR-29b and miR-125a-5p to be *bona fide* targets of EGFR signaling (significantly regulated by EGF treatment and reversed by gefitinib). Among these candidates, miR-125a-5p appeared to be particularly intriguing, because its level was altered most significantly by EGF stimulation (Fig. 1C), and it has been shown that miR-125a regulates the phosphorylation of ERK1/2 and Akt in breast cancer cells [6].

### MicroR-125a-5p negatively regulated cell migration and invasion

**Fig. 2:**
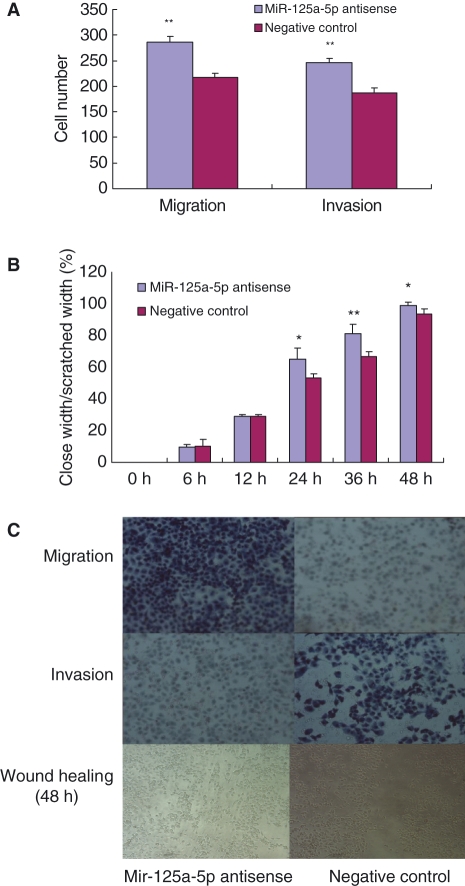
Promotional effects of antisense miR-125a-5p on migration and invasion of PC9 cells. (A) Assay of migration and invasion of antisense miR-125a-5p across 8 μm porous membranes relative to negative control. (B) Confluent cell monolayers were wounded with a pipette tip. Wound closure was monitored by microscopy at the indicated times. Data are given as closed width/scratched width (%). (C) Representative photomicrographs of migration, invasion and wound-healing in PC9 cells were taken with a Nikon ECLIPSETS 100 microscope. **P < 0.001 and *P < 0.005, as compared with the negative control. Magnification: for migration and invasion, ×200; for wound-healing, ×100.

EGFR signaling has been shown to play an important role in cell migration and invasion [15]. Thus, the marked repression of miR-125a-5p after EGFR activation prompted us to investigate whether miR-125a-5p influenced tumor metastasis. We first performed Transwell cell migration assays. PC9 cells were selected as a model system with which to assess the function of miR-125a-5p, because they expressed endogenous miR-125a-5p at a relatively high level before EGF stimulation (Fig. 1C). Our results showed that treatment with antisense miR-125a-5p could significantly increase cell motility (Fig. 2A). To examine their invasion capability, cells transfected with antisense miR-125a-5p or negative control were plated on top of a layer of extracellular matrix (ECM) extracted from mouse sarcoma. Consistent with the migration results, knockdown of miR125a-5p significantly promoted invasion (Fig. 2A). To further determine the function of miR125a-5p in cell migration, we tested the polarized migration of cells by a wound-healing assay. As shown in Fig. 2B, PC9 cells transfected with antisense miR125a-5p healed the scratch wound much faster than the negative control. Representative photographs of migration, invasion and wound-healing are shown in Fig. 2C. Taken together, our data pointed to an important role of miR-125a-5p in regulating cell migration and invasion, suggesting that it might regulate the metastasis of lung cancer.

### Inhibition of miR-125a-5p increased cell survival and tube formation

**Fig. 3:**
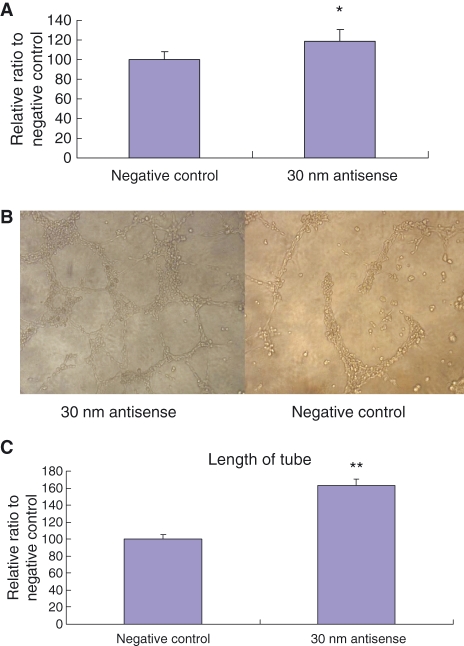
Antisense miR-125a-5p facilitated the growth of PC9 cells and tube formation of ECV304 cells. (A) PC9 cells (5 × 10^3^) cells were plated on 96-well plates. Forty-eight hours later, MTT was added to each well for 3 h at 37 °C, and then replaced by dimethylsulfoxide. Absorbance was read at 570 nmol·L^−1^. The data are presented as percentage of growth relative to the negative control. (B) ECV304 cells were cultured in a 12-well plate coated with ECM gel. Photographs of tube formation were taken using a Nikon ECLIPSETS 100 microscope (under ×200 magnification). (C) Total tube length was measured with image analysis software. **P < 0.001, and *P* < 0.005, as compared with the negative control.

In addition to regulating migration and invasion, EGFR signaling also influences proliferation, angiogenesis, apoptosis, and cell cycle progression [15]. After finding that miR-125a-5p negatively regulated cell migration and invasion, we went on to determine whether miR-125a-5p had an impact on cell proliferation, angiogenesis, apoptosis, and cell cycle progression. We first examined its potential function in cell proliferation, which contributes heavily to tumor development. A comparison with mock-transfected cells showed that antisense miR-125a-5p significantly enhanced PC9 cell growth (Fig. 3A).

To answer the question of whether miR-125a-5p is also involved in angiogenesis, we treated ECV304 cells, showing significant expression of endogenous miR-125a-5p (data not shown), with antisense miR-125a-5p. After culture, angiogenesis was assessed with tube formation assays. Consistent with its potential tumor-suppressing role, we found that knockdown of miR-125a-5p significantly enhanced the tube formation efficiency of ECV304 cells (Fig. 3B,C).

Subsequently, we performed fluorescence-activated cell sorting (FACS)-based cell cycle profiling and apoptosis analysis. However, miR-125a-5p antisense did not influence apoptosis or the cell cycle of PC9 cells (data not shown). Taken together, our results demonstrated that miR-125a-5p played an inhibitory role in lung cancer metastasis.

### Decreased miR-125a-5p expression in a subset of human lung cancers

To gain further insights into the role of miR-125a-5p in lung carcinogenesis and to examine the clinical relevance of our findings, we investigated the expression of miR-125a-5p in a panel of lung cancer patient samples together with paired counterpart normal tissues. With the criterion of a 2^−ΔΔCt^ value change of no less than 2 between the malignant and normal groups, we found that 33.33% (5/15) of lung cancer samples showed significantly decreased expression of miR-125a-5p by real-time RT-PCR. Thus, our results suggested that downregulation of miR-125a-5p might contribute, at least partially, to lung cancer development in human patients.

## Discussion

The EGFR signal transduction pathway regulates essential cellular functions, and appears to play a central role in the etiology and progression of numerous epithelial malignancies, including lung cancer [4]. Moreover, the function of EGFR mutations in survival of lung cancer patients and clinical respones to gefitinib has been reported [16,17]. Thus, the identification and characterization of potential factors that regulate EGFR pathways are critical to our understanding of lung cancer development and progression.

Emerging evidence has revealed the profound role of various miRNAs in regulating cancer development. Different miRNAs have been implicated in the formation of neoplasms, malignant progression, and metastasis. To examine the potential miRNA targets of EGFR signaling, we used the miRHuman_10.0_070802 miRNA array, and identified miR-125a-5p as being regulated by EGFR activation. To examine the cellular function of miR-125a-5p, we employed comprehensive *in vitro* approaches to establish the inhibitory role of miR-125a-5p in cell proliferation, angiogenesis, cell motility, and invasion. It is also of great interest and importance to note that about one-third of the human lung cancer samples that we examined revealed significant downregulation of miR-125a-5p expression. Consistent with our findings, miR-125a-5p has also been reported to be downregulated in breast cancer biopsy specimens [18,19]. Further investigations will be performed to determine whether miR-125a-5p expression is clinically correlated with lung cancer metastasis. Interestingly, the present results, which showed miR-125a-5p negatively regulating cancer cell metastasis, are consistent with our previous work, which suggested that miR-125a-5p is negatively correlated with lung cancer metastasis [11]. Together, the findings presented here strongly suggest that miR-125a-5p may function as a tumor suppressor.

Except for let-7i, miR-24, miR-25, miR-29b, and miR-125a-5p, our miRNA array analysis also indicated another 42 miRNAs with significant differences by comparing miRNA expression before and after gefitinib treatment (*P* < 0.01; Table S2). Interestingly, among them, some miRNAs, such as miR-16, miR-143, miR-200b, and miR-205, were shown to be involved in human cancer [8,20,21].

In view of our findings here and the results of Scott *et al.* [6], showing that miR-125a blocked ERK1/2 and Akt signaling in breast cancer cells, we will determine whether miR-125a-5p regulates the phosphorylation of Akt and/or ERK1/2 in lung cancer cells, and whether miR-125a-5p downregulates ErbB2 and ErbB3 in lung cancer cells, because the present work only focused on the functional analysis of miR-125-a-5p.

In conclusion, we identified miR-125a-5p, an EGFR-regulated miRNA, as a potential tumor metastasis suppressor. Our results further substantiated the role of miRNA in tumorigenesis, and revealed the possibility of using miRNAs as potential therapeutic targets to specifically suppress oncogenic signaling pathways that go awry in human cancers.

## Experimental procedures

### Cells and cultures

The human lung cancer cell line A549 was obtained from the American Type Culture Collection (Manassas, VA, USA) and maintained in Ham’s F12K medium (Invitrogen, Carlsbad, CA,USA) supplemented with 10% fetal bovine serum (Shanghai Sangon Biological Engineering Technology and Services Co., Ltd, Shanghai, China). The human lung cancer cell lines H1299 and PC-9 were obtained from Zhejiang Cancer Hospital and grown in RPMI-1640 medium (Invitrogen) supplemented with 10% fetal bovine serum. Human umbilical vein endothelial cells (ECV-304) were obtained from the China Center for Type Culture Collection (Wuhan, China), and cultured in RPMI-1640 medium supplemented with 10% fetal bovine serum.

### Drugs and chemicals

Recombinant human EGF was purchased from Invitrogen. Gefitinib (AstraZeneca, Macclesfield, UK) was a gift from D. Chunfeng (Zhejiang Hospital, Hangzhou, China). A 250 mg gefitinib tablet was dissolved in 25 mL of dimethylsulfoxide and stored at −20 °C. Antibody against EGFR and antibody against phospho-EGFR were purchased from Cell Signaling Technology (Beverly, MA, USA). Antibodies against ERK1/2 and phospho-ERK1/2 were from Chemicon International, Inc. (CA, USA). Antibody against Akt was from BioVision, Inc. (CA, USA), and antibody against phospho-Akt was from Santa Cruz Biotechnology, Inc. (Santa Cruz, CA, USA).

### Western blot analysis

To examine the influence of gefitinib on phosphorylation of proteins, confluent tumor cells were pretreated with gefitinib at 0, 1, 2, 5 and 10 μm for 2 h before exposure to EGF (20 ng·mL^−1^) for 30 min at 37 °C. The cells were then rinsed with ice-cold NaCl/P_i_, and lysed in chilled lysis buffer comprising 10 mm Tris/HCl (pH 7.4), 1% NP-40, 0.1% deoxycholic acid, 0.1% SDS, 150 mm NaCl, 1 mm EDTA, and 1% Protease Inhibitor Cocktail (Sigma, CA, USA). Protein concentrations were measured using the Bio-Rad protein assay (Bio-Rad Laboratories, San Jose, CA, USA), according to the manufacturer’s instructions. Then, 30 μg portions of cell lysates were subjected to SDS/PAGE and transferred to Immobilon membranes (Millipore, Bedford, MA, USA). After transfer, the blots were incubated with blocking solution, probed with various antibodies, and washed. Proteins were detected using goat anti-(rabbit IgG) (MultiSciences Biotech Co., Ltd, Hangzhou, China). β-Actin (Anti-beta-Actin Monoclonal Antibody; MultiSciences Biotech Co., Ltd, Hangzhou, China) was used as a positive control.

### RNA isolation and miRNA microarray

On the day after subculturing, cells were cultured under different conditions for 48 h: serum-starved medium, serum-starved medium plus EGF (20 ng·mL^−1^), and serum-starved medium plus 1 μm (PC9) or 10 μm (A549 and H1299) gefitinib plus EGF (20 ng·mL^−1^). RNA was extracted with Trizol reagent (Invitrogen) as the standard method. Separation, quality control, labeling, hybridization and scanning of small RNA were performed by LC Sciences (Houston, TX, USA), using the miRHuman_10.0_070802 miRNA array chip, based on Sanger miRBase Release 10.0. Preliminary statistical analysis was performed by LC Sciences on raw data normalized by the locally weighted scatterplot smoothing (LOWESS) method on the background-subtracted data. Then, in-depth data analysis was performed to identify EGFR-regulated miRNA expression. MicroRNA with *P* < 0.01 was considered as having a significant difference.

### Real-time quantitative RT-PCR

Reverse transcription reactions were carried out using dNTP, Moloney murine leukemia virus reverse transcriptase and RiboLock ribonuclease inhibitor (Applied Biosystems, Foster City, CA, USA). Real-time PCR was performed on an ABI PRISM 7300 Sequence Detection System (Applied Biosystems), using an SYBR Green I Real-Time PCR kit (GenePharma, Shanghai, China) for miR-24, miR-25, miR-29b, and miR-125a-5p, and TaqMan Universal PCR Master Mix, No AmpErase UNG (Applied Biosystems) for let-7i; 5s and RUN6B (Applied Biosystems) were used as positive controls. The relative expression levels of miRNAs in each sample were calculated and quantified by using the 2^−ΔΔCt^ method after normalization for expression of positive control. Primers for reverse transcription and PCR are given in Table S1.

### Cell migration and invasion assay

We performed the Transwell insert (24-well insert; pore size, 8 μm; Corning, Inc., Corning, NY, USA) assay to evaluate PC9 cell migration and invasion *in vitro*. In both the migration assay and the invasion assay, an initial equilibrium, obtained by adding 0.6 mL of RPMI-1640 with 10% fetal bovine serum to the multiple-well plate, was employed to enhance cell attachment. For the invasion assay, the inserts were coated with extracellular matrix gel from Engelbreth–Holm–Swarm mouse sarcoma (Sigma, Santa Clara, CA, USA). On the following day, 1 × 10^5^ cells suspended in 0.1 mL of fresh medium without fetal bovine serum were added to the insert. Forty-eight hours after seeding, the cells on the upper surface of the membrane were removed using cotton buds. Cell monolayers on the lower surface of the insert were fixed and stained using standard cytological techniques. Six visual field of each insert were randomly counted under a microscope (using 10 × 20 lenses).

### Wound-healing experiment

Cells (1 × 010^6^) were seeded on six-well plates. Upon confluence, the cell layer was scratched with a P-200 pipette tip (Qiagen, Valencia, CA, USA) and then grown in normal conditions after being washed with culture medium. Photographs of the wound adjacent to reference lines scraped on the bottom of the plate were taken using a Nikon ECLIPSE TS100 microscope (under ×100 magnification), and the wound-healing was measured at 0, 6, 12, 24, 36 and 48 h, respectively. Sextuple assays were performed for each experiment. Data were described as closed width/scratched width (%, mean ± standard deviation).

### Tube formation assay

ECV304 cells (1 × 10^5^) were cultured in a 12-well plate coated with 200 μL of ECM gel. Photographs of the tube formation were taken using a Nikon ECLIPSETS 100 microscope (under ×200 magnification), and the length of tube was quantified with image analysis software (developed at the US National Institutes of Health, and available on the Internet at http://rsb.info.nih.gov/nih-image/). Each experiment was repeated three times.

### Cell proliferation assay

A Vybrant 3-(4,5-dimethylthiazol-2-yl)-2,5-diphenyl-tetrazolium bromide (MTT) Cell Proliferation Assay Kit (Invitrogen) was used to estimate the effect of antisense miR-25a-5p on the proliferation of PC9 cells. Cells were seeded at a density of 5 × 10^3^ cells per well in 96-well plates. After incubation for 48 h, 20 μL of MTT solution (5 mg·mL^−1^ in NaCl/P_i_) was added to each well for 3 h at 37 °C. Subsequently, culture medium with MTT was removed, and formazan crystals were reabsorbed in 200 μL of dimethylsulfoxide (Shanghai Sangon Biological Engineering Technology and Services Co., Ltd, Shanghai, China). Absorbance was read at 570 nmol·L^−1^ using a Universal Microplate Spectrophotometer (Bio-tek Instruments, Inc., Winooski, VT, USA). Each experiment was performed in six replicate wells. Values for control cells were considered as 100% viability.

### Apoptosis analysis

PC9 cells were seeded in six-well plates (1 × 10^6^ cells per well). Seventy-two hours post-transfection, the cells were harvested and stained with fluorescein isothiocyanate-conjugated antibody against annexin V and propidium iodide, using the annexin V–fluorescein isothiocyanate apoptosis detection kit (B. D. Biosciences Pharmingen, San Jose, CA, USA). Stained cells were then quantified by FACSCalibur flow cytometry (Becton Dickinson, Sandy, UT, USA).

### Cell cycle detection

PC9 cells were plated in six-well plates (1 × 10^6^ cells per well). Seventy-two hours post-transfection, the adherent cells and the supernatants were collected and centrifuged at 500 ***g*** for 10 min. Cell pellets were washed with NaCl/P_i_ containing BSA (Invitrogen), fixed using 70% methanol, and stored at −20 °C. The fixed cells were subsequently washed twice with NaCl/P_i_ and stained with PI and RNase A (B. D. Biosciences Pharmingen), and DNA content was analyzed by flow cytometry (Becton Dickinson).

### Human lung cancer samples

Primary human lung cancers and paired noncancerous normal lung samples were obtained from 15 patients treated at the Zhejiang Province Cancer Hospital, with documented informed consent being obtained in each case. Samples were frozen in liquid nitrogen and stored at −80 °C until use. RNA extraction and quantitative RT-PCR were performed as above.

### Transfection

Cells were transfected with the Pre-miRTM miRNA Precursor of miR-125a-5p (Ambion, Inc., Austin, TX, USA) and Anti-miR miRNA Inhibitors of miR-125a-5p (Ambion) at 30 nmol·L^−1^ final concentration, using NeoFx (Ambion). Efficiency of transfection was confirmed by real-time RT-PCR. CyTM3-labeled Pre-miRTM Negative Control#1 (Ambion) and Anti-miR Negative Control#1 (Ambion) were used as negative controls.

### Statistical methods

Differences between groups were compared using Pearson’s chi-square test for qualitative variables and Student’s *t*-test for continuous variables. *P* < 0.05 was considered to be significant.

## Abbreviations

ECM: extracellular matrix
EGF: epidermal growth factor
EGRF: epidermal growth factor receptor
ERK: extracellular signal-related kinase
FACS: fluorescence-activated cell sorting
miRNA: microRNA
MTT: 3-(4,5-dimethylthiazol-2-yl)-2,5-diphenyl-tetrazolium bromide

## Supporting information

The following supplementary material is available:

**Table S1.** Primers used in RT-PCR.

**Table S2.** Additional miRNAs responding to gefitinib treatment in lung cancer cells (*P* < 0.01).

This supplementary material can be found in the online article.

Please note: As a service to our authors and readers, this journal provides supporting information supplied by the authors. Such materials are peer-reviewed and may be re-organized for online delivery, but are not copy-edited or typeset. Technical support issues arising from supporting information (other than missing files) should be addressed to the authors.
