# Supplementary material for: Epidermal growth factor receptor-regulated miR-125a-5p – a metastatic inhibitor of lung cancer
Source: FEBS J. 2009 Oct;276(19):5571–8. doi: 10.1111/j.1742-4658.2009.07238.x (PMC2776928; doi:10.1111/j.1742-4658.2009.07238.x)
Supplement: Supplementary file 1 [file ejb0276-5571-SD1.pdf]

Additional file Table S1 Primers of RT and PCR used in RT-PCR.

RNA name	RTprimer	Forward primer	Backward primer
let-7i	ABI	ABI	ABI
miR-24	5'GTCGTATCCAGTGCG TGTCGTGGAGTCGGCA ATTGCACTGGATACGA CCTGTTCC 3'	5'TGTCGTGG AGTCGGCAA T 3'	5'TGGCTC AGTTCAG CAGGA3'
miR-25	5'GTCGTATCCAGTGCG TGTCGTGGAGTCGGCA ATTGCACTGGATACGA CTCAGCC 3'	5'AGTGCGTG TCGTGGAGT CGG 3'	5'CATTGC ACTTGTC TCGGTCT G3'
miR-29b	5'GTCGTATCCAGTGCG TGTCGTGGAGTCGGCA ATTGCACTGGATACGA CAACACTG 3'	5'AGTGCGT GTCGTGGA GT 3'	5'TAGCA CCATTTG AAATCAG T3'
miR-125a-5p	5'GTCGTATCCAGTGCA GGGTCCGAGGTATTCG CACTGGATACGACTCA CAGG3'	5'GGGTCCG AGGTATTCG CACT3'	5'TCCCTG AGACCCT TTAACCT GTG3'
5S	5'GGCCCGACCCTGCT TAG3'	5'TCTACGGC CATACCACC	5'GGCCC GACCCTG

		CTGAA3’	CTTAG3’
RUN6B	ABI	ABI	ABI

Additional file Table S2 Additional miRNAs responding to gefitinib treatment in lung cancer cells (P<0.01)

Name	Un-treated	Gefitinib treatment	Log ratio
miR-127-3p	51	881	4.39
miR-376c	20	1 047	4.13
miR-495	28	392	3.51
miR-494	55	1 080	3.46
miR-376a	14	170	3.35
miR-379	39	670	3.24
miR-487b	34	704	3.23
miR-654-3p	48	169	3.18
miR-143	24	359	2.8
miR-134	49	509	2.79
miR-329	19	187	2.79
miR-299-5p	27	197	2.46
miR-29c	792	1 124	1.55
miR-22	746	1 053	0.46
miR-100	9 420	6 234	-0.28
miR-31	6 505	5 403	-0.48

miR-107	3 352	1 952	-0.72
miR-151-3p	1 277	702	-0.79
miR-185	677	440	-0.80
miR-425	740	388	-0.82
miR-103	3 997	2 178	-0.87
miR-191	5 309	2 538	-0.88
miR-28-5p	512	242	-0.98
miR-320	8 351	4 238	-1.05
miR-132	628	325	-1.09
miR-16	11 899	6 345	-1.10
miR-92a	13 630	6 233	-1.30
miR-423-5p	3 117	1 336	-1.29
miR-20a	6 759	2 432	-1.30
miR-194	909	253	-1.31
miR-200b	2 695	579	-1.35
miR-98	359	118	-1.65
miR-93	3 214	969	-1.74
miR-92b	6 099	1 699	-1.93
miR-374b	958	220	-2.07
miR-205	1 288	1 780	-2.30
miR-877	438	78	-2.39
miR-584	1 020	190	-2.42

miR-454	624	71	-2.87
miR-203	743	73	-3.36
miR-182	4 666	1 005	-3.66
miR-196a	178	18	-5.88
